# Src homology domain-containing phosphatase 2 suppresses cellular senescence in glioblastoma

**DOI:** 10.1038/bjc.2011.345

**Published:** 2011-09-20

**Authors:** L-M Sturla, P O Zinn, K Ng, M Nitta, D Kozono, C C Chen, E M Kasper

**Affiliations:** 1 Department of Neurosurgery, Beth Israel Deaconess Medical Center, Boston, MA, USA; 2 Department of Radiation Oncology, Dana-Farber Cancer Institute, Boston, MA, USA; 3 Department of Neurosurgery, Tokyo Women's Medical University, Tokyo, Japan

**Keywords:** glioblastoma, phosphatases, SHP2, senescence

## Abstract

**Background:**

Epidermal growth factor receptor (EGFR) signalling is frequently altered during glioblastoma *de novo* pathogenesis. An important downstream modulator of this signal cascade is SHP2 (Src homology domain-containing phosphatase 2).

**Methods:**

We examined the The Cancer Genome Atlas (TCGA) database for SHP2 mutations. We also examined the expression of a further 191 phosphatases in the TCGA database and used principal component and comparative marker analysis available from the Broad Institute to recapitulate the TCGA-defined subgroups and identify the specific phosphatases defining each subgroup. We identified five siRNAs from two independent commercial sources that were reported by the vendor to be pre-optimised in their specificity of SHP2 silencing. The specificity and physiological effects of these siRNAs were tested using an *in vitro* glioma model.

**Results:**

TCGA data demonstrate SHP2 to be mutated in 2% of the glioblastoma multiforme's studied. Both mutations identified in this study are likely to be activating mutations. We found that the four subgroups of GBM as defined by TCGA differ significantly with regard to the expression level of specific phosphatases as revealed by comparative marker analysis. Surprisingly, the four subgroups can be defined solely on the basis of phosphatase expression level by principal component analysis. This result suggests that critical phosphatases are responsible for the modulation of specific molecular pathways within each subgroup. Src homology domain-containing phosphatase 2 constitutes one of the 12 phosphatases that define the *classical subgroup*. We confirmed the biological significance by siRNA knockdown of SHP2. All five siRNAs tested reduced SHP2 expression by 70–100% and reduced glioblastoma cell line growth by up to 80%. Profiling the established molecular targets of SHP2 (ERK1/2 and STAT3) confirmed specificity of these siRNAs. The loss of cell viability induced by SHP2 silencing could not be explained by a significant increase in apoptosis alone as demonstrated by terminal deoxyribonucleotidyl transferase-mediated nick-end labelling and propidium iodide staining. Src homology domain-containing phosphatase 2 silencing, however, did induce an increase in *β*-galactosidase staining. Propidium iodide staining also showed that SHP2 silencing increases the population of glioblastoma cells in the G1 phase of the cell cycle and reduces the population of such cells in the G2/M- and S-phase.

**Conclusion:**

Src homology domain-containing phosphatase 2 promotes the growth of glioblastoma cells by suppression of cellular senescence, a phenomenon not described previously. Selective inhibitors of SHP2 are commercially available and may be considered as a strategy for glioblastoma therapy.

Glioblastoma multiforme (GBM) is the most common type of malignant primary brain tumour in adults. Around 30 000 new cases are diagnosed every year in the United States and Europe (CBTRUS, 2008). The prognosis is dismal, and despite treatment with the standard of care regimen involving surgery, radiation 'and chemotherapy, median survival remains below 15 months (Stupp *et al*, 2005), and there is a clear need for improved therapeutic approaches. There has, however, been substantial progress in the understanding of molecular cancer subgroups (Verhaak *et al*, 2010), pathways involved in gliomagenesis and disease progression (Furnari *et al*, 2007; Network CGAR, 2008; Parsons *et al*, 2008; Yan *et al*, 2009; Verhaak *et al*, 2010) These efforts to understand the underlying molecular biology of the disease is now paving the way for the development of targeted therapeutics.

Epidermal growth factor receptor (EGFR) is overexpressed in a variety of human tumours including GBM, where it has been linked to radiation resistance and poor prognosis. A number of researchers, including us, have shown activation of EGFR to result in cytoprotective and proliferative downstream signalling (Schmidt-Ullrich *et al*, 2003; Sturla *et al*, 2005). Studies of receptor tyrosine kinases (RTKs), including EGFR, demonstrate that the overall phosphorylation state is a net result of RTK and protein tyrosine phosphatase (PTP) activities (Reynolds *et al*, 2003). As the catalytic activity of PTP's can be 1000-fold greater than that of kinases (Haque *et al*, 1995; Wang *et al*, 2010), perturbation of activity may have a significantly more profound effect on signal propagation than that of kinases. Most targeting strategies for RTKs emphasise the kinase activity (Shimizu *et al*, 2008). In autocrine-regulated tumour cells, the Tyr kinase activity is always ‘on’, and thus net RTK activity will be mostly regulated by PTP activity. This suggests that the greatest therapeutic gain may be achieved by targeting the counteracting PTP.

Src homology domain-containing phosphatase 2 (SHP2) (PTPN11) is a non-receptor PTP, which regulates several of the RTK pathways known to be overexpressed in glioblastoma, including EGFR, FGFR and PDGFR (Grossmann *et al*, 2010). Perhaps, the most well-studied role of SHP2 is that in the modulation of EGFR phosphorylation, the RTK most widely overexpressed in GBM (Bredel *et al*, 2010). Here SHP2 has been shown to both antagonise and potentiate the action of its target PTK's and was the first phosphatase described as oncogenic (Bentires-Alj *et al*, 2004). More than 58 different SHP2 mutations have been identified in various tumours and 18 mutations in *Noonan* and *Leopard* syndromes, where patients exhibit disruption of normal cell proliferation and migration during development (Bentires-Alj *et al*, 2004). In its basal state, SHP2 activity is suppressed by intramolecular interactions between residues in the ‘backside loop’ of the N-terminal SH2 domain and the catalytic surface of the PTP domain (Hof *et al*, 1998). The mutations have been found to cluster mostly in the N-SH2 and PTP domain interface of the protein and are therefore predicted to be activating mutations – suggesting a positive role for SHP2 in tumourigenesis

As mutations in SHP2 have been identified in a variety of solid tumours and this phosphatase is an important regulator of multiple RTK's involved in the aetiology of GBM, we decided to examine this phosphatase closely in both established GBM cell lines and the The Cancer Genome Atlas (TCGA) human tissue database. We used both TCGA mutation and expression data to confirm the presence of SHP2 mutations in human GBM and establish a potential role for this phosphatase in the classical, RTK-driven subgroup. We also used an siRNA approach to examine the effects of SHP2 knockdown on cell viability in established GBM cell lines *in vitro*.

## Materials and methods

### TCGA analysis

Median expression data for 207 GBM samples from two different microarray platforms combined was downloaded from the TCGA data portal. Sample data were rearranged to group tumours into the four subclasses defined by TCGA (Verhaak *et al*, 2010). The resulting file was converted to a gct file compatible with the GenePattern software available from the Broad Institute (http://www.broadinstitute.org/). Files were generated from this master file containing either the 189 phosphatase genes available on the microarray platforms or the 189 phosphatase genes combined with the 693 known kinase genes allowing a comparison to the whole genome. Principal component analysis (PCA) was performed on both the phosphatase and whole genome data sets using the gene pattern software.

The GenePattern software was used to perform K nearest-neighbour analysis (Sun *et al*, 2011) using leave-one-out cross-validation (KNN X-validation). This class prediction analysis was used to determine how accurately the samples could be grouped into their various classes using only phosphatase expression as compared to the whole genome.

Gene pattern comparative marker analysis (CMA) was used to identify significant markers for each group using only phosphatase genes, phosphatase and kinase genes combined or the entire gene set. Marker phosphatases were then fed into both the pathway interaction database (http://pid.nci.nih.gov/) and ingenuity pathway analysis (http://www.ingenuity.com/) to look for enrichment of pathways in each group.

### Cell lines and tissue culture

Established glioblastoma cell lines, U87 and A172, were obtained from the ATCC and maintained in Dulbecco's modified Eagle's medium high glucose with Penstrep and 15% FBS (Invitrogen, Carlsbad, CA, USA) at 37 °C, 5% CO_2_.

### Transfection with siRNA

Cells were cultured to a confluency of 30% before siRNA transfection. Following this siRNA was transfected into cells using Lipofectamine RNAiMAX (Invitrogen). Transfection was carried out according to the manufacturer's instructions using a final concentration of 10 nM siRNA. Transfection efficiency was assessed using a fluorescent non-targeting siRNA (Allstars Negative siRNA Alexafluor488; Qiagen, Valencia, CA, USA). The non-targeting control siRNA (Allstars Negative siRNA) and SHP2-specific siRNAs Hs_PTPN11_7 (Q1; SI02225909) and Hs_PTPN11_6 (Q2; SI02225902) were also purchased from Qiagen. Stealth RNAi-negative control low GC duplex and high GC duplex and the SHP2-specific siRNA PTPN11HSS108832 (I1), PTPN11HSS108833 (I2) and PTPN11HSS108834 (I3) were purchased from Invitrogen.

### Western blotting and antibodies

Cells were rinsed with ice-cold phosphate-buffered saline (PBS) and snap-frozen on dry ice at the appropriate times following transfection. Cells were scraped into a denaturing cell extraction buffer (Invitrogen) containing a protease inhibitor cocktail (Pierce, Rockford, IL, USA) and 100 *μ*g ml^−1^ PMSF. Lysates were incubated for 30 min on ice and passed five times through a 20-gauge needle and syringe. Samples were centrifuged at 14 000 *g* for 15 min at 4 °C, and supernatant protein concentrations were determined by the Bradford assay (Biorad, Hercules, CA, USA). For whole-cell lysates, 5 × loading buffer (50 mM NaPO_4_, 5% SDS, 0.25% bromophenol blue, 12.5% 2-mercaptoethanol and 10% glycerol) was added to lysates to achieve 1 × . Equal amounts of protein were fractionated on SDS/10% polyacrylamide gels and protein transferred electrophoretically onto nitrocellulose membranes. Membranes were probed with the appropriate primary and secondary antibodies. Blots were analysed by chemiluminescence detection (Supersignal West Pico; Pierce). The SHP2 antibody was used at a dilution of 1:500; phospho- and total STAT3 dilution was 1:500 and phospho- and total ERK1/2 was 1 : 1000 in 5% milk TBS-T. Equality of protein loading was confirmed by *β*-actin staining 1 : 50 000 in 5% milk TBS-T. All antibodies, with the exception of SHP2 and *β*-actin-HRP (Abcam, Cambridge, UK), were purchased from Cell Signaling Technology (Danvers, MA, USA). The HRP-conjugated anti-rabbit and anti-mouse antibodies were diluted 1 : 5000 in 5% milk TBS-T. Blots were stripped with Restore Western Blot Stripping Buffer (Pierce), and re-probed as described in the text.

### Cell viability assay

Cells were cultured in 10 cm plates and transfected with 10 nM appropriate siRNAs. At 24 h following transfection, cells were trypsinised and reseeded at a density of 1000 cells per well in a 96-well plate, 8 wells per condition. Outer rows were not used and media were changed every 3 days to avoid assay error due to media evaporation. At 6 and 10 days post-transfection, alamar blue (Invitrogen) was added at a concentration of 10% per well and cells incubated for 3 h at 37 °C, 5% CO_2_. Colorimetric change (fluorescence) was assessed using a Biorad plate reader, Ex 570 nm and Em 585 nm.

### *β*-Galactosidase assay

Cells cultured and transfected for viability assay were seeded in triplicates at a density of 1000 cells per well in 24-well plates at the time of re-seeding for viability assay. Cells were incubated at 37 °C for 4 days following reseeding and stained for *β*-galactosidase, using a *β*-galactosidase staining kit according to the manufacturer's instructions (Cell Signaling Technology). Plates were viewed and photographed using a Zeiss inverted microscope, × 20 magnification.

### TUNEL and cell cycle analysis

Cells were seeded in 10 cm plates and transfected as described previously with 10 nM of the appropriate SHP2 siRNAs or the death control siRNA (Qiagen). Cells were harvested by trypsinisation, rinsed twice in PBS and fixed overnight in 70% ice-cold ethanol. Cells were rinsed in PBS and assessed for DNA degradation by terminal deoxyribonucleotidyl transferase-mediated nick-end labelling (TUNEL) according to the manufacturer's instructions (Apoptag; Intergen, Burlington, MA, USA). Analysis of cell cycle was determined as stated above, but following fixation, cells were incubated with RNAse (100 μg ml^−1^; Invitrogen) for 30 min before propidium iodide labelling (25 μg ml^−1^; Roche, Indianapolis, IN, USA). Terminal deoxyribonucleotidyl transferase-mediated nick-end labelling positivity and propidium iodide staining was determined by flow cytometry.

## Results

### GBM harbours SHP2 mutations likely to be activating mutations

**Figure 1:**
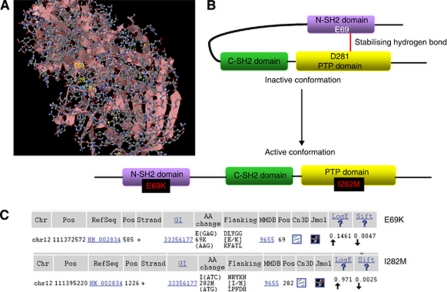
The Cancer Genome Atlas mutation data reveal potential activating mutations of SHP2 in glioblastoma. The TCGA mutation database was searched for all mutations of SHP2. Only those mutations validated in tumour *vs* normal are shown here. (**A**) Structural representation of SHP2 showing the mutations found in GBM, which cluster in the N-SH2 and PTP domain interfaces. (**B**) Schematic representation of SHP2. (**C**) Mutation data for validated SHP2 mutations identified by TCGA and validated against paired normal tissue.

The TCGA identified four mutations of SHP2 in tumour normal matched GBM samples. Two of these mutations were validated and found to be present in tumour samples only (Figure 1). The first mutation is a single base mutation, resulting in a glutamic acid to lysine change at position 69. This amino acid is situated within the N-terminal SH2 domain and forms a stabilising hydrogen bond with aspartic acid 281 in the PTP domain. The mutation, originally identified in the neuroblastoma, was copied *in vitro* and found to have a 16-fold higher phosphatase activity than wild-type SHP2 (Bentires-Alj *et al*, 2004). The N-SH2 domain sits in the catalytic site of the PTP domain inhibiting SHP2 phosphatase activity. Not surprisingly, mutations in the N-SH2 domain have been found to significantly increase SHP2 phosphatase activity (Bentires-Alj *et al*, 2004).

The second mutation results in an isoleucine to methionine substitution at position 282 within the PTP domain. This is adjacent to the 281 aspartic acid residue involved in the hydrogen bond shown to stabilise the inactive conformation of SHP2. Although this mutation has not previously been described, its location suggests that it could also be an activating mutation.

The third mutation results in a leucine to histidine substitution at position 262 also within the PTP domain. The final mutation is a threonine to methionine substitution at position 553 within the c-terminal domain. However, these final two mutations were not validated as tumour-specific.

### SHP2 is a marker of the classical subgroup of GBM

**Figure 2:**
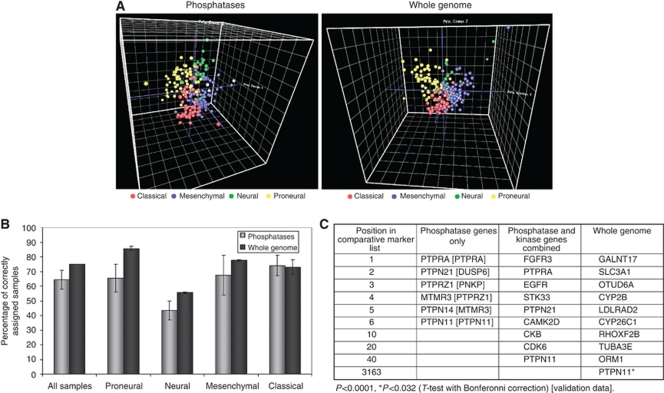
Analysis of TCGA profiling data reveal potential role of SHP2 in defining the classical subgroup of GBM. (**A**) Principal component analysis of TCGA GBM data using 191 phosphatase genes or the whole genome. (**B**) K nearest-neighbour analysis using leave-one-out cross-validation (KNN X-validation) and the most significant 10 features (most differentially expressed genes) were used to determine how accurately the samples could be grouped into their various classes using only phosphatase expression *vs* the whole genome. Data are shown for both test and validation data sets as the mean percentage correctly assigned samples±s.e.m. There was no significant difference between the percentage of correctly assigned samples determined using phosphatase expression only and the comparative value as determined using the whole genome (unpaired *t*-test, *P*=0.17–0.92). (**C**) Comparative marker analysis identified most significant genes that define the classical subgroup of GBM using phosphatase genes only, phosphatase and kinase genes combined or the whole gene expression profile. Validation data results are shown within square parenthesis [ ].

We found that the four subgroups of GBM, as defined by TCGA, differ significantly with regard to the expression level of specific phosphatases. The four subgroups can be defined solely on the basis of phosphatase expression by PCA (Figure 2A).

The gene pattern software was used to perform K nearest-neighbor analysis using leave-one-out cross-validation. This class prediction analysis was used to determine how accurately the samples could be grouped into their various classes using only phosphatase expression as compared to the combined phosphatase/kinase or whole genome. K nearest-neighbour cross-validation using only phosphatase gene expression accurately predicted the sample class for 64.5±6.5% of the samples when analysis was performed on both the original and validation data sets. This compares to 75±0% when using the whole genome (Figure 2B). When the accuracy of prediction is broken down by class, however, the phosphatase-only gene set accurately predicts the sample class for 74±7% of the classical samples compared to 73±5% using the whole genome. There was no significant difference between percentage correctly assigned samples determined using phosphatase expression only and that determined using the whole genome (unpaired *t*-test, *P*=0.17–0.92).

Comparative marker analysis identified SHP2 as one of the 12 phosphatases that define the classical subgroup (Figure 2C). In all, 12 phosphatases were found to be significantly associated with classical GBM, with SHP2 being sixth on the list with a *P*-value <0.0001. When CMA was used with the phosphatase and kinase genes combined, SHP2 fell to 40th in the list, but retained significance with a *P*-value <0.0001. Finally when CMA was carried out with the entire gene expression profile of over 20 000 genes, SHP2 falls to the 3163rd position with a *P*-value of 0.032. Src homology domain-containing phosphatase 2 therefore holds its significance as a classical subgroup-defining phosphatase even in the context of the entire gene set. When this analysis was repeated using a validation data set available from TCGA, SHP2 remained as a significant marker of the classical subgroup (Figure 2C).

### SHP2 siRNA decreases GBM cell viability without a significant increase in apoptosis

**Figure 3:**
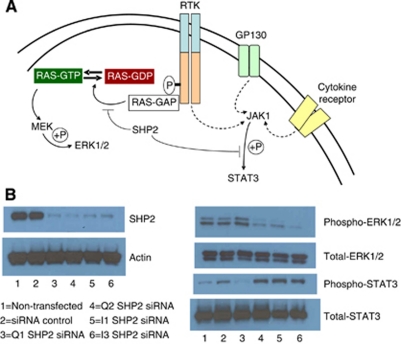
Specificity of SHP2 siRNA was confirmed by western blot of well-characterised downstream targets of SHP2, ERK1/2 andSTAT3. (**A**) SHP2 dephosphorylates RAS-GAP binding sites on RTK's, resulting in enhanced RAS activity and consequent MAPK phosphorylation. Src homology domain-containing phosphatase 2 negatively regulates STAT3 phosphorylation. (**B**) Specificity of SHP2-specific siRNA was shown by determination of the phosphorylation status of the SHP2 targets ERK1/2 and STAT3 3–6 days following transfection with 10 nM siRNA.

All five siRNAs tested reduced SHP2 expression by 70–100% as compared to the non-targeting siRNA or a mock-transfected control from 36 h post-transfection (Figure 3). Equality of protein loading was confirmed by actin staining. Profiling the phosphorylation status of established molecular targets of SHP2 (ERK1/2 and STAT3) (Agazie and Hayman, 2003; Zhang *et al*, 2009) confirmed specificity of these siRNAs. Src homology domain-containing phosphatase 2 has previously been shown to enhance Ras activation by dephosphorylating EGFR tyrosine 992, responsible for the translocation of RAS GAP to the plasma membrane, where it inhibits RAS activity (Agazie and Hayman, 2003). Knockdown of SHP2 by SHP2-specific siRNA resulted in a decrease in ERK1/2 phosphorylation without a significant change in total ERK1/2 levels as shown by western blot. In contrast, SHP2 has been shown to have an inhibitory effect on JAK-STAT signalling (Zhang *et al*, 2009). In accordance with the established effect of SHP2 on STAT3, knockdown of SHP2 siRNA resulted in an increase in phospho-STAT3 without a significant increase in total STAT3 levels (Figure 3).

**Figure 4:**
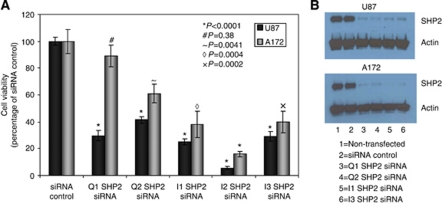
Small interfering RNA knockdown of SHP2 reduces viability of glioblastoma cell lines. (**A**) U87 and A172 cells were transfected with 10 nM siRNA and cell viability was assessed 10 days following transfection using an alamar blue assay. (**B**) Knockdown of SHP2 protein levels was confirmed by western blot.

The viability of U87 and A172 cells transfected with non-targeting siRNA was not significantly different to that of untransfected or mock-transfected cells (data not shown). Viability of U87 and A172 cells transfected with SHP2-specific siRNA reduced glioblastoma cell line growth by up to 80%, as shown using an alamar blue assay (Figure 4). Five commercially available SHP2-specific siRNAs were tested. Of these, one siRNA had a much greater effect on cell viability than the others, siRNA I2, and was eliminated from further study in case of off-target toxic effects. Viability of U87 cells was reduced by 60–75% by the four remaining siRNAs (*P*<0.0001). A172 cell viability was reduced by up to 60% (*P*<0.005).

**Figure 5:**
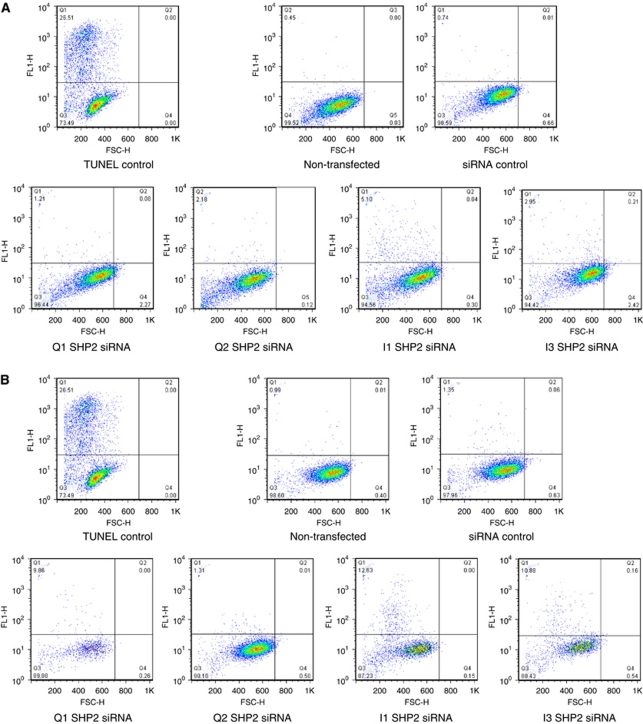
Reduced U87 and A172 cell viability cannot be accounted for by an increase in cellular apoptosis. Apoptosis was assessed by TUNEL and flow cytometry 4 days following transfection of (**A**) U87 cells and (**B**) A172 cells with 10 nM SHP2-specific siRNA.

Reduced cell viability could not be accounted for by enhanced apoptosis as shown by TUNEL. The classical DNA degradation associated with apoptosis was assessed by TUNEL in non-transfected, non-targeting siRNA-transfected and SHP2-specific siRNA-transfected cells. Less than 1% of the control cell populations were shown to be apoptotic as determined by TUNEL positivity (Figure 5). U87 cells transfected with either Q1 or Q2 SHP2-specific siRNA exhibited TUNEL positivity in 1% and 2% of the cell population, while I1 and I3 showed 5% and 3%, respectively (Figure 5A). Although the percentage of apoptotic cells was higher in SHP2 siRNA-transfected cells as compared to the non-transfected and non-targeting siRNA controls, this was not high enough to account for the loss of cell viability observed. Similarly in A172 cells, only 1% of the cell population was TUNEL positive. Although this percentage increased to 10%, 13% and 11% in Q1, I1 and I3 SHP2-specific siRNA-transfected cells, respectively, this was not high enough to explain reduced cell viability (Figure 5B).

### SHP2 siRNA induces cellular senescence

**Figure 6:**
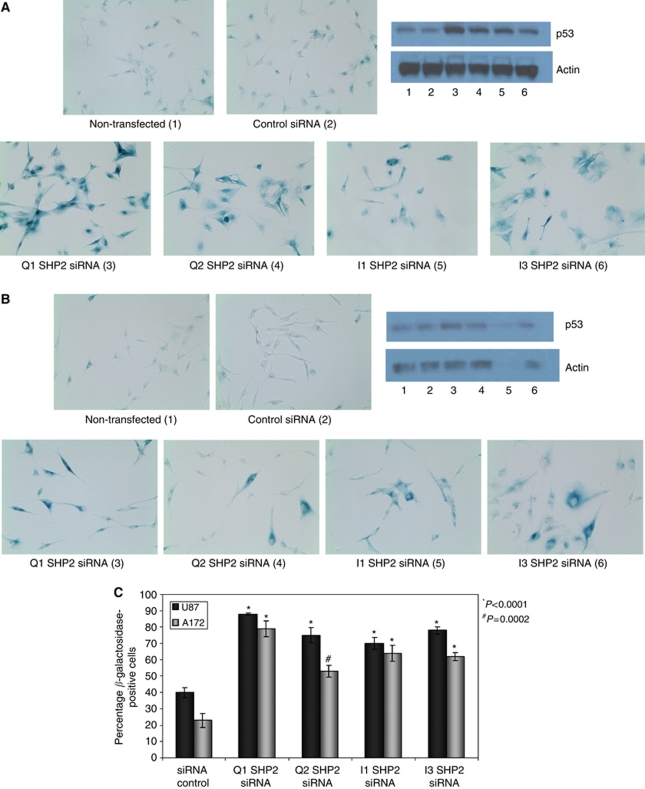
SHP2-specific siRNA induces senescence in U87 and A172 cells. Senescence was determined by the classic morphological changes, including enlarged and flattened or elongated morphology and *β*-galactosidase staining in (**A**) U87 and (**B**) A172 cells transfected with 10 nM control or SHP2-specific siRNA 4 days following transfection. Western blot was used to determine p53 level. Images were captured at a magnification of × 20. (**C**) Cells with >15% positivity as determined using the ImageJ software (NIH) were considered positive for quantitation. Two fields per replicate and a total of 2000 cells were counted. Data are shown as percentage-positive cells±s.e.m.

Both U87 and A172 cells transfected with SHP2-specific siRNA showed significant morphological changes. Cells showed enlarged and flattened or elongated morphology consistent with that seen in senescent cells (Figure 6). Although similar morphological changes were noted in U87 cells (Zhan *et al*, 2009), cellular senescence was neither studied or suggested as a mechanism.

As we saw a loss in cell viability without a significant increase in cellular apoptosis alongside morphological changes consistent with senescence, we further analysed the effects of SHP2 knockdown on cellular senescence. Cells with knockdown of SHP2 exhibited cellular enlargement and elongation classically associated with senescence (Figure 6). *β*-Galactosidase staining at pH 6.0 is also a classical marker of cellular senescence (Figure 6). Src homology domain-containing phosphatase 2 knockdown induced a significant increase in both intensity of *β*-galactosidase staining and number of *β*-galactosidase-positive cells in both U87 (Figure 6A) and A172 cells (Figure 6B) from 4 days post-transfection. Cells with >15% positivity as determined using the ImageJ software (NIH) were considered positive for quantitation. Two fields per replicate and a total of 2000 cells were counted. Knockdown of SHP2 induced a highly significant increase in the percentage of *β*-galactosidase-positive cells in both U87 and A172 cells as compared to a control siRNA or mock-transfected control (Figure 6C). Cell populations transfected with SHP2 siRNA demonstrated 60–90% *β*-galactosidase positivity as compared to 20–40% *β*-galactosidase positivity in cells transfected with the negative control siRNA (*P*<0.0001). An increase in p53 protein level, as determined by western blot, was also observed in the majority of SHP2 knockdown cells. This is consistent with the increase in p53 known to precede cellular senescence (Quick and Gewirtz, 2006). The increase in p53 level was inconsistent in Figure 6B (lane 5) due to limited sample. The actin loading control is also reduced in this lane. Owing to the drastic effect of SHP2 knockdown on cell viability, it is not always possible to get sufficient protein for western blot following viability and senescence assays.

Propidium iodide staining was also used to observe cell populations in the various phases of the cell cycle. Senescent cells have previously been shown to arrest in the late G1 phase of the cell cycle, and consistent with this phenomenon, we found an increase in the percentage of cells in the G1 phase of the cell cycle in SHP2 knockdown cells (data not shown).

## Discussion

Our analysis of TCGA data showed that phosphatase expression alone can be used to recapitulate the subdivision of GBM into four subgroups as identified by TCGA using the whole genome-derived 840 gene identifier (Verhaak *et al*, 2010), suggesting that phosphatases play an important role in the underlying biology of GBM. As kinases are far more numerous than phosphatases, one phosphatase must target multiple kinases. As such, targeting phosphatases may prove more effective in tumours, such as GBM, with co-activation of multiple RTK pathways, which respond poorly to kinase inhibitor monotherapy.

We identified activating mutations of the putative oncogenic phosphatase, SHP2, in approximately 2% of the tumours analysed by TCGA. Both of the mutations that passed validation in the TCGA data set could be considered activating mutations based on their location within the N-SH2/PTP domain interface. The first mutation, E69K, has been well studied and is known to have 16-fold higher phosphatase activity than wild-type SHP2 (Bentires-Alj *et al*, 2004), although a more recent study by the same group determined that the tumourigenic potential of SHP2 mutation does not necessarily correlate with PTP activity (Keilhack *et al*, 2005). With respect to the second mutation, we predict this to be an activating mutation due to its location adjacent to a hydrogen bond known to stabilise the inactive conformation.

We also considered the expression profile of phosphatases in the four GBM subgroups described by TCGA. Comparative marker analysis identifying the phosphatases most significantly associated with each group clearly identified SHP2 as an important marker of the classical subgroup of GBM and remained a significant marker even when considered alongside kinases or the whole genome. Considering the well-documented role for SHP2 in the regulation of EGFR phosphorylation, it is not too surprising that the classical subgroup of glioblastoma, as defined by TCGA, was also the group found to have deregulation of EGFR signalling. High-level EGFR amplification was observed in 97% of this subtype along with 70% of the EGFRvIII mutations (Verhaak *et al*, 2010). Platelet-derived growth factor subunit A, also overexpressed in this group, has been shown to enhance EGFR signalling through heterodimerisation of PDGFRA and EGFR (Milenkovic *et al*, 2003).

Src homology domain-containing phosphatase 2 is well known to extend the half-life of active RAS and increase ERK1/2 signalling downstream of a variety of RTKs, including EGFR, PDGFR and FGFR, all of which have been found to be overexpressed in GBM (Giannini *et al*, 2005).

The classical subgroup of glioblastoma, as defined by TCGA, was found to have high expression of EGFR and PDGFA. Platelet-derived growth factor subunit A has been shown to enhance EGFR signalling through heterodimerisation of PDGFRA and EGFR (Milenkovic *et al*, 2003). High-level EGFR amplification was also observed in 97% of this subtype along with five of seven EGFRvIII mutations (Verhaak *et al*, 2010). Given that SHP2 has been shown by several groups to regulate EGFR activity (Wu *et al*, 2000; Grossmann *et al*, 2010), it is not surprising that we also found SHP2 to be a significant marker of the classical subgroup of GBM.

Given the association of SHP2 expression with the RTK-driven ‘classical’ subgroup of GBM and the identification of potential activating mutations in GBM samples, it is not surprising that knockdown of SHP2 with SHP2-targeting siRNA resulted in a loss of cell viability. This loss in viability, however, was not associated with a significant increase in apoptosis. We did see striking morphological changes in the cells with significant knockdown of SHP2. These changes were similar to those described as classical cellular senescence. Although the morphological changes observed in our study were noted in a previous study, no connection to cellular senescence was made (Zhan *et al*, 2009). We found various markers of senescence when we knocked down SHP2 expression in GBM cell lines, suggesting that SHP2 protects the cells from senescence. It is therefore possible that the presence of activating mutations of SHP2 in GBM allows tumour progression by protecting cells from senescence and apoptosis. The mechanism of action is unknown at present, but two strong possibilities exist. Src homology domain-containing phosphatase 2 is well known to enhance the half-life of active RAS and as such it is possible that the knockdown of SHP2 activity and its consequent effects on RAS activity are responsible for the senescence that we observe in GBM cell lines. We confirmed a reduction in the MAPKs, ERK1/2, downstream of RAS in cells with reduced SHP2 protein levels. Although Ras is an important mediator of glioblastoma tumourigenesis, its loss is not typically associated with the induction of cellular senescence. In fact, there are several reports of induction of senescence by overexpression of active RAS, a phenomenon known as oncogene-induced senescence (Rai *et al*, 2010; Kosar *et al*, 2011). Previous studies have also shown SHP2-activating mutations to be mutually exclusive to mutations such as NF1 loss, which leads to an increase in active RAS (Holzel *et al*, 2010).

Src homology domain-containing phosphatase 2 has been shown, however, to regulate hTERT localisation (Jakob *et al*, 2008). The human telomerase catalytic subunit – when localised to the nucleus – protects cells against cellular senescence. Human telomerase reverse transcriptase alone has been found to immortalise normal human astrocytes (Sonoda *et al*, 2001) with the addition of SV40 T-Ag and active Ras (H-ras), allowing maximum tumourigenicity as determined by anchorage-independent growth and formation of tumours in nude mice (Rich *et al*, 2001). This is consistent with the finding that most grade III gliomas express telomerase. Its reactivation is associated with non-malignant grade II to malignant grade III conversion (Kim *et al*, 1994; Sano *et al*, 1998). A study by Jakob *et al* (2008) showed overexpression of SHP2 to block oxidative stress-induced nuclear export of hTERT. As a consequence, hTERT is retained in the nucleus, resulting in resistance to cellular senescence and apoptosis. Preliminary data using an hTERT antibody and IHC (data not shown) suggest that knockdown of SHP2 expression using siRNA reduces nuclear hTERT staining in U87 cells. More work is required to confirm these data and to determine the exact mechanism by which SHP2 suppresses cellular senescence in glioblastoma, but it is clear that it plays an important role in the viability of these cells. As the selectivity of commercially available SHP2 inhibitors is improved, they should be considered a potential strategy for glioblastoma therapy.

## References

[bib1] Agazie YM, Hayman MJ (2003) Molecular mechanism for a role of SHP2 in epidermal growth factor receptor signaling. Mol Cell Biol 23: 7875–7886 14560030 10.1128/MCB.23.21.7875-7886.2003 PMC207628

[bib2] Bentires-Alj M, Paez JG, David FS, Keilhack H, Halmos B, Naoki K, Maris JM, Richardson A, Bardelli A, Sugarbaker DJ, Richards WG, Du J, Girard L, Minna JD, Loh ML, Fisher DE, Velculescu VE, Vogelstein B, Meyerson M, Sellers WR, Neel BG (2004) Activating mutations of the Noonan syndrome-associated SHP2/PTPN11 gene in human solid tumors and adult acute myelogenous leukemia. Cancer Res 64: 8816–8820 15604238 10.1158/0008-5472.CAN-04-1923

[bib3] Bredel M, Scholtens DM, Yadav AK, Alvarez AA, Renfrow JJ, Chandler JP, Yu IL, Carro MS, Dai F, Tagge MJ, Ferrarese R, Bredel C, Phillips HS, Lukac PJ, Robe PA, Weyerbrock A, Vogel H, Dubner S, Mobley B, He X, Scheck AC, Sikic BI, Aldape KD, Chakravarti A, Harsh GR (2010) NFKBIA deletion in glioblastomas. N Engl J Med 364: 627–637 21175304 10.1056/NEJMoa1006312 PMC3652611

[bib4] CBTRUS (2008) Statistical Report: Primary Brain Tumors in the United Status, 2000–2004. In Central Brain Tumor Registry of the United States (http://www.CBTRUS.org)

[bib5] Furnari FB, Fenton T, Bachoo RM, Mukasa A, Stommel JM, Stegh A, Hahn WC, Ligon KL, Louis DN, Brennan C, Chin L, DePinho RA, Cavenee WK (2007) Malignant astrocytic glioma: genetics, biology, and paths to treatment. Genes Dev 21: 2683–2710 17974913 10.1101/gad.1596707

[bib6] Giannini C, Sarkaria JN, Saito A, Uhm JH, Galanis E, Carlson BL, Schroeder MA, James CD (2005) Patient tumor EGFR and PDGFRA gene amplifications retained in an invasive intracranial xenograft model of glioblastoma multiforme. Neuro-oncology 7: 164–176 15831234 10.1215/S1152851704000821 PMC1871885

[bib7] Grossmann KS, Rosario M, Birchmeier C, Birchmeier W (2010) The tyrosine phosphatase Shp2 in development and cancer. Adv Cancer Res 106: 53–89 20399956 10.1016/S0065-230X(10)06002-1

[bib8] Haque SJ, Flati V, Deb A, Williams BR (1995) Roles of protein-tyrosine phosphatases in Stat1 alpha-mediated cell signaling. J Biol Chem 270: 25709–25714 7592750 10.1074/jbc.270.43.25709

[bib9] Hof P, Pluskey S, Dhe-Paganon S, Eck MJ, Shoelson SE (1998) Crystal structure of the tyrosine phosphatase SHP-2. Cell 92: 441–450 9491886 10.1016/s0092-8674(00)80938-1

[bib10] Holzel M, Huang S, Koster J, Ora I, Lakeman A, Caron H, Nijkamp W, Xie J, Callens T, Asgharzadeh S, Seeger RC, Messiaen L, Versteeg R, Bernards R (2010) NF1 is a tumor suppressor in neuroblastoma that determines retinoic acid response and disease outcome. Cell 142: 218–229 20655465 10.1016/j.cell.2010.06.004 PMC2913027

[bib11] Jakob S, Schroder P, Lucosz M, Spyridopoulos I, Altschmeid J, Haendler J (2008) Nuclear protein tyrosine phosphatase Shp-2 is one important negative regulator of nuclear export of telomerase reverse transcriptase. J Biol Chem 283: 33155–33161 18829466 10.1074/jbc.M805138200 PMC2662250

[bib12] Keilhack H, David FS, McGregor M, Cantley LC, Neel BG (2005) Diverse biochemical properties of Shp2 mutants: implications for disease phenotypes. J Biol Chem 280(35): 30984–30993 15987685 10.1074/jbc.M504699200

[bib13] Kim NW, Piatyszek MA, Prowse KR, Harley CB, West MD, Ho PL, Coviello GM, Wright WE, Weinrich SL, Shay JW (1994) Specific association of human telomerase activity with immortal cells and cancer. Science 266: 2011–2015 7605428 10.1126/science.7605428

[bib14] Kosar M, Bartkova J, Hubackova S, Hodny Z, Lukas J, Bartek J (2011) Senescence-associated heterochromatin foci are dispensable for cellular senescence, occur in a cell type- and insult-dependent manner, and follow expression of p16 (ink4a). Cell Cycle 10: 457–468 21248468 10.4161/cc.10.3.14707

[bib15] Milenkovic I, Weick M, Wiedemann P, Reichenbach A, Bringmann A (2003) P2Y receptor-mediated stimulation of Muller glial cell DNA synthesis: dependence on EGF and PDGF receptor transactivation. Invest Ophthalmol Vis Sci 44: 1211–1220 12601051 10.1167/iovs.02-0260

[bib16] Network CGAR (2008) Comprehensive genomic characterization defines human glioblastoma genes and core pathways. Nature 455: 1061–1068 18772890 10.1038/nature07385 PMC2671642

[bib17] Parsons DW, Jones S, Zhang X, Lin JC, Leary RJ, Angenendt P, Mankoo P, Carter H, Siu IM, Gallia GL, Olivi A, McLendon R, Rasheed BA, Keir S, Nikolskaya T, Nikolsky Y, Busam DA, Tekleab H, Diaz Jr LA, Hartigan J, Smith DR, Strausberg RL, Marie SK, Shinjo SM, Yan H, Riggins GJ, Bigner DD, Karchin R, Papadopoulos N, Parmigiani G, Vogelstein B, Velculescu VE, Kinzler KW (2008) An integrated genomic analysis of human glioblastoma multiforme. Science (New York, NY) 321: 1807–1812 10.1126/science.1164382 PMC2820389 18772396

[bib18] Quick QA, Gewirtz DA (2006) An accelerated senescence response to radiation in wild-type p53 glioblastoma multiforme cells. J Neurosurg 105: 111–118 16871885 10.3171/jns.2006.105.1.111

[bib19] Rai P, Young JJ, Burton DG, Giribaldi MG, Onder TT, Weinberg RA (2010) Enhanced elimination of oxidized guanine nucleotides inhibits oncogenic RAS-induced DNA damage and premature senescence. Oncogene 30: 1489–1496 21076467 10.1038/onc.2010.520

[bib20] Reynolds AR, Tischer C, Verveer PJ, Rocks O, Bastiaens PI (2003) EGFR activation coupled to inhibition of tyrosine phosphatases causes lateral signal propagation. Nat Cell Biol 5: 447–453 12717446 10.1038/ncb981

[bib21] Rich JN, Guo C, McLendon RE, Bigner DD, Wang XF, Counter CM (2001) A genetically tractable model of human glioma formation. Cancer Res 61: 3556–3560 11325817

[bib22] Sano T, Asai A, Mishima K, Fujimaki T, Kirino T (1998) Telomerase activity in 144 brain tumours. Br J Cancer 77: 1633–1637 9635839 10.1038/bjc.1998.267 PMC2150049

[bib23] Schmidt-Ullrich RK, Contessa JN, Lammering G, Amorino G, Lin PS (2003) ERBB receptor tyrosine kinases and cellular radiation responses. Oncogene 22: 5855–5865 12947392 10.1038/sj.onc.1206698

[bib24] Shimizu M, Shirakami Y, Moriwaki H (2008) Targeting receptor tyrosine kinases for chemoprevention by green tea catechin, EGCG. Int J Mol Sci 9: 1034–1049 19325845 10.3390/ijms9061034 PMC2658783

[bib25] Sonoda Y, Ozawa T, Hirose Y, Aldape KD, McMahon M, Berger MS, Pieper RO (2001) Formation of intracranial tumors by genetically modified human astrocytes defines four pathways critical in the development of human anaplastic astrocytoma. Cancer Res 61: 4956–4960 11431323

[bib26] Stupp R, Mason WP, van den Bent MJ, Weller M, Fisher B, Taphoorn MJ, Belanger K, Brandes AA, Marosi C, Bogdahn U, Curschmann J, Janzer RC, Ludwin SK, Gorlia T, Allgeier A, Lacombe D, Cairncross JG, Eisenhauer E, Mirimanoff RO (2005) Radiotherapy plus concomitant and adjuvant temozolomide for glioblastoma. N Engl J Med 352: 987–996 15758009 10.1056/NEJMoa043330

[bib27] Sturla LM, Amorino G, Alexander MS, Mikkelsen RB, Valerie K, Schmidt-Ullrichr RK (2005) Requirement of Tyr-992 and Tyr-1173 in phosphorylation of the epidermal growth factor receptor by ionizing radiation and modulation by SHP2. J Biol Chem 280: 14597–14604 15708852 10.1074/jbc.M413287200

[bib28] Sun X, Mei S, Tao H, Wang G, Su L, Jiang S, Deng C, Xiong Y, Li F (2011) Microarray profiling for differential gene expression in PMSG-hCG stimulated preovulatory ovarian follicles of Chinese Taihu and Large White sows. BMC Genom 12: 111 10.1186/1471-2164-12-111 PMC3047302 21324170

[bib29] Verhaak RG, Hoadley KA, Purdom E, Wang V, Qi Y, Wilkerson MD, Miller CR, Ding L, Golub T, Mesirov JP, Alexe G, Lawrence M, O’Kelly M, Tamayo P, Weir BA, Gabriel S, Winckler W, Gupta S, Jakkula L, Feiler HS, Hodgson JG, James CD, Sarkaria JN, Brennan C, Kahn A, Spellman PT, Wilson RK, Speed TP, Gray JW, Meyerson M, Getz G, Perou CM, Hayes DN (2010) Integrated genomic analysis identifies clinically relevant subtypes of glioblastoma characterized by abnormalities in PDGFRA, IDH1, EGFR, and NF1. Cancer Cell 17: 98–110 20129251 10.1016/j.ccr.2009.12.020 PMC2818769

[bib30] Wang V, Davis DA, Veeranna RP, Haque M, Yarchoan R (2010) Characterization of the activation of protein tyrosine phosphatase, receptor-type, Z polypeptide 1 (PTPRZ1) by hypoxia inducible factor-2 alpha. PLoS One 5: e9641 20224786 10.1371/journal.pone.0009641 PMC2835759

[bib31] Wu CJ, Chen Z, Ullrich A, Greene MI, O’Rourke DM (2000) Inhibition of EGFR-mediated phosphoinositide-3-OH kinase (PI3-K) signaling and glioblastoma phenotype by signal-regulatory proteins (SIRPs). Oncogene 19: 3999–4010 10962556 10.1038/sj.onc.1203748

[bib32] Yan H, Parsons DW, Jin G, McLendon R, Rasheed BA, Yuan W, Kos I, Batinic-Haberle I, Jones S, Riggins GJ, Friedman H, Friedman A, Reardon D, Herndon J, Kinzler KW, Velculescu VE, Vogelstein B, Bigner DD (2009) IDH1 and IDH2 mutations in gliomas. N Engl J Med 360: 765–773 19228619 10.1056/NEJMoa0808710 PMC2820383

[bib33] Zhan Y, Counelis GJ, O’Rourke DM (2009) The protein tyrosine phosphatase SHP-2 is required for EGFRvIII oncogenic transformation in human glioblastoma cells. Exp Cell Res 315: 2343–2357 19427850 10.1016/j.yexcr.2009.05.001 PMC2724964

[bib34] Zhang W, Chan RJ, Chen H, Yang Z, He Y, Zhang X, Luo Y, Yin F, Moh A, Miller LC, Payne RM, Zhang ZY, Fu XY, Shou W (2009) Negative regulation of Stat3 by activating PTPN11 mutants contributes to the pathogenesis of Noonan syndrome and juvenile myelomonocytic leukemia. J Biol Chem 284: 22353–22363 19509418 10.1074/jbc.M109.020495 PMC2755958

